# Biogenic Volatile Organic Compounds from Pennsylvanian Lakes Sampled during the 2024 Algal Bloom Season

**DOI:** 10.1021/acsestair.5c00240

**Published:** 2025-11-04

**Authors:** Christine Troller, Dallan Schoenberger, Richard Spear, Jamie Detweiler, Jeffery Butt, Coty N. Jen

**Affiliations:** † Department of Chemical Engineering, Carnegie Mellon University, 5000 Forbes Ave, Pittsburgh, Pennsylvania 15213, United States; ‡ Center for Atmospheric Particle Studies, Carnegie Mellon University, 5000 Forbes Ave, Pittsburgh, Pennsylvania 15213, United States; § Department of Environmental Protection, Southwest Regional Office, 400 Waterfront Drive, Pittsburgh, Pennsylvania 15222, United States; ∥ Department of Environmental Protection, Bureau of Clean Water, Rachel Carson State Office Building, 400 Market Street, Harrisburg, Pennsylvania 17101, United States

**Keywords:** Harmful algal blooms, freshwater gas flux, ammonia, amines, pyrroline, pyridine

## Abstract

Biogenic volatile organic compounds (BVOCs) emitted from aquatic systems are increasingly recognized for their influence on atmospheric chemistry. However, emissions from freshwater environments, specifically during harmful algal bloom (HAB) events, remain poorly quantified. These HAB events are increasing globally in frequency and intensity, driven by climate change, nutrient runoff, and land-use changes. This study investigates the water-to-air gas exchange rates of BVOCs from southwestern Pennsylvanian freshwater lakes during peak HAB conditions, with a focus on nitrogen-containing compounds that are typically underrepresented in atmospheric measurements. Using atmospheric pressure, hydronium chemical ionization mass spectrometry (CIMS), we measured BVOC emissions from 18 lake samples in the laboratory, capturing real-time fluxes of 900 unique masses. Ammonia, pyrroline, and pyridine consistently exhibited the highest emission fluxes across samples. Alkylamines were less abundant, although they remain atmospherically important due to their role in new particle formation. These results represent the first reported real-time freshwater flux measurements of alkylamines, pyrroline, and pyridine, offering new insight into the atmospheric implications of HABs. Notably, no clear correlation was observed between BVOC fluxes and chlorophyll *a* and phycocyanin concentrations, which were taken to represent the cyanobacterial concentrations in the samples. This suggests that emissions are influenced by other biological or chemical factors not captured in this study. Principal component analysis identified two significant outlier water samples, driven by elevated ammonia, pyrroline, and an unknown compound at 124.101 amu, though the underlying cause of these deviations remains unresolved. The remaining lake emissions were similar. These findings provide foundational observations to better understand the role of freshwater system emissions in regional air quality and atmospheric processes.

## Graphical abstract

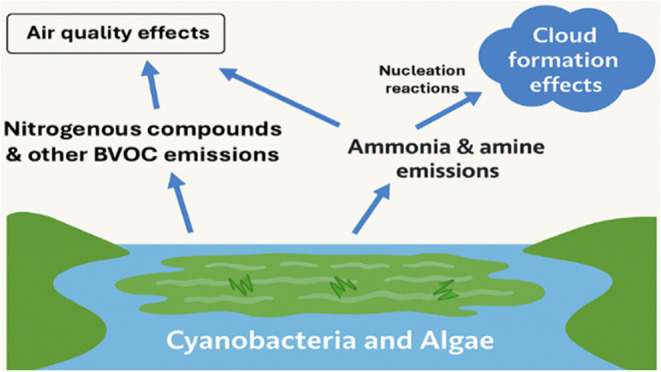

## Introduction

Biogenic volatile organic compounds (BVOCs) play a significant role in atmospheric chemistry by participating in new particle formation and secondary organic aerosol production. These processes influence regional air quality, in addition to cloud formation and cloud properties. A wide variety of previously studied BVOCs (e.g., isoprene, monoterpenes, alcohols) are emitted from terrestrial and aquatic sources and can be highly influenced by environmental stressors.^1–5^ However, many additional BVOCs are emitted from environmental sources but remain poorly characterized, especially in aquatic environments where measurements are limited. BVOCs pose potential health risks, particularly when oxidized in the atmosphere into more reactive or toxic products such as ozone and fine particulate matter, which are associated with respiratory and cardiovascular issues.^6–9^ Additionally, certain BVOCs (e.g., alkylamines and nitrogen-containing heterocycles), are themselves known to be irritating or toxic when inhaled.^10,11^ Despite their impacts, the current understanding of sources, fluxes, and chemical transformations of BVOCs remains incomplete, particularly outside of well-studied terrestrial environments. Expanding our knowledge of BVOC sources is essential for improving air quality forecasting and for understanding feedback between ecosystems and the atmosphere.

While aquatic ecosystems are recognized sources of BVOCs,^5^ much of the current literature focuses on the marine environment due to the ocean’s vast spatial coverage. However, BVOCs produced by freshwater systems, such as lakes and rivers, can influence regional atmospheric chemistry, particularly in areas with high population density or concentrated agricultural activity. While a few studies suggest that freshwater lakes emit a wide range of BVOCs, such as nonmethane hydrocarbons and oxygenated BVOCs, these emissions remain under-characterized.^12–14^ Limited research has characterized BVOC production from lakes and rivers, but highlights the potential importance of these sources^13,15–17^ A study conducted on Chaohu Lake in China demonstrated that cyanobacterial blooms potentially enhanced the emission of various BVOCs (e.g., alkanes, alkenes, aromatics, alcohols, aldehydes).^12^ These emissions have the potential to contribute to atmospheric photochemical reactions and regional air quality issues. Given that freshwater environments are often located near human populations and are susceptible to environmental change, there is a clear need to further investigate and quantify their BVOC emissions.

In aquatic environments, BVOCs are produced by diverse microbial communities, including phytoplankton, bacteria, and cyanobacteria, as well as through inputs from terrestrial runoff. Microbial activity is a primary driver of BVOC emissions, with studies demonstrating that emissions correlate with chlorophyll *a* (chl-a) concentrations and overall biomass in marine and freshwater environments.^12,18,19^ The diversity of microbial life in freshwater lakes and rivers likely results in a complex array of emitted BVOCs, many of which vary in composition and quantity depending on microbial community structure, nutrient availability, and environmental stressors. As such, BVOC emissions from aquatic microbes are expected to scale with microbial abundance and may shift significantly during algal bloom events. In addition to biological factors, the presence of a surfactant microlayer, typically composed of organic material, lipids, and microbial exudates, may also modulate the BVOC exchange at the water–air interface.^20^

Freshwater microbial communities are highly responsive to changes in water chemistry, often experiencing more rapid and pronounced shifts than marine systems due to the smaller size of freshwater bodies and their direct exposure to surrounding land use and climate variability.^21^ These shifts are increasingly manifested as visible algal blooms (see Figure 1), particularly harmful algal blooms (HABs), which are becoming more frequent and more intense on a global scale.^22^ The median annual algal bloom frequency in freshwater lakes has increased by approximately 1.8% each year over the past two decades.^23^ Additionally, satellite analyses reveal that peak summertime phytoplankton bloom intensity has increased in 68% of 71 large lakes globally over the past 30 years, suggesting a widespread intensification of algal bloom events.^24^ These blooms, often dominated by cyanobacteria, are typically associated with toxin production, oxygen depletion, and significant ecological disruption.^25–27^ Given the strong link between microbial biomass and BVOC production, it is expected that emissions from freshwater HABs may scale with bloom biomass, representing an important but poorly quantified component of freshwater and atmospheric interactions.

**Figure 1:**
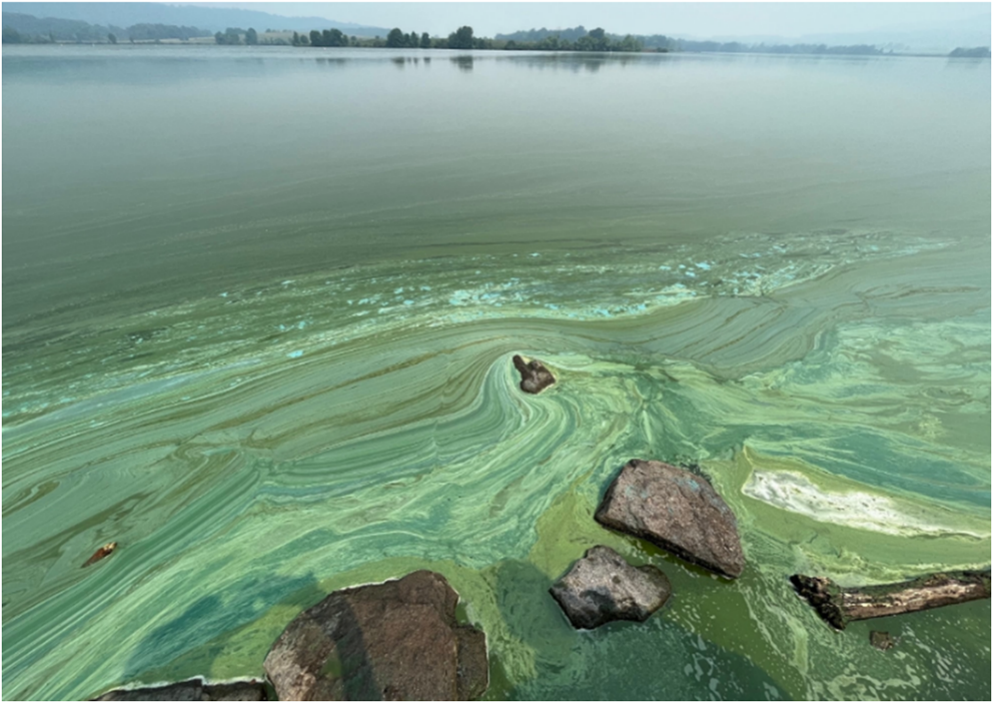
Harmful algal bloom, Middle Creek Lake, Stevens, PA, 6/8/23. Photo courtesy of Jeffery Butt (PA DEP).

Cyanobacteria release ammonia and alkylamines (e.g., methylamine, dimethylamine, trimethylamine) into aquatic systems, where these compounds undergo water-to-air exchange and contribute to atmospheric new particle formation and other atmospheric processes, impacting cloud properties and Earth’s radiative balance.^10^ Furthermore, ammonia and alkylamines are highly reactive in the atmosphere, with typical lifetimes ranging from a few hours to a few days depending on the environmental conditions.^28^ These compounds readily react with acidic species (e.g., sulfuric acid and nitric acid) and atmospheric oxidants such as hydroxyl radicals, contributing to formation of secondary organic particles and air pollution.^10^ However, only limited ammonia and alkylamine flux estimates have been reported, primarily from marine environments.^29–33^ Cyanobacteria also release nitrogen-containing heterocycles such as pyrroline and pyridine, compounds known to interact with biological systems and, in some cases, exhibit toxicity or carcinogenicity. Only one study observed pyrroline in freshwater cyanobacterial emissions,^34^ while pyridine derivatives and other aromatic compounds have been observed in various cyanobacterial biological processes.^10,35^ Furthermore, cyanobacteria release many other unidentified and unmeasured compounds, making their full impact on atmospheric and air quality processes unknown.

The purpose of this study is to quantify the water-to-air gas exchange rate (i.e., flux) of BVOCs from freshwater lakes during peak harmful algal bloom conditions, with a focus on compounds that exhibit high proton affinity using an atmospheric pressure hydronium chemical ionization mass spectrometer (CIMS). CIMS offers enhanced sensitivity, on the parts per quadrillion (ppqv) level,^36,37^ to nitrogen-containing BVOCs such as ammonia, alkylamines, and organic heterocycles, which are compounds often missed in traditional proton transfer mass spectrometer (PTR) measurements. However, some PTR inlet designs, such as shortened sampling tubes that are heated, have been deployed to measure nitrogen-containing BVOCs.^38,39^

We report gaseous flux measurements of BVOCs from 18 lake samples collected from freshwater lakes in southwestern Pennsylvania between July and November 2024. Specifically, ammonia, alkylamines, pyrroline, and pyridine were examined, but over 900 unique masses were observed. Note that some of these masses are of compounds clustered with water or other compounds. Ammonia, pyrroline, and pyridine were among the top ten highest fluxes measured across all lake sample emissions, emphasizing their importance in understanding the broader environmental impacts of freshwater sources. Although the alkylamine fluxes were not among the top ten highest fluxes observed, their emissions into the atmosphere play an outsized role in new particle formation. In addition, an untargeted principal component data analysis on all measured emission compounds was performed to determine sample variability and provide a more comprehensive understanding of the lake sample emissions.

Combined, this study represents the first reported real-time measurements of freshwater alkylamine, pyrroline, and pyridine BVOC fluxes from HABs. These observations provide valuable baseline data for future focus on the role of alkylamines and heterocyclic amines in water-to-air exchange.

## Methods

### Lake Sample Collection

Eighteen 16 L freshwater lake samples were collected from seven different lake locations in southwestern PA between July and November 2024 (See Table 1 for lake GPS location, location type, and sample collection dates). The lakes ranged from urban, suburban, to rural locations and thus likely experienced different types of land runoff and human activity.

**Table 1:** Sample Collection Lakes with Corresponding GPS Locations, Type, and Collection Dates

lake	GPS location/type	collection dates
Burrell Lake	40.580043, −79.686396/suburban	7/10, 7/24, 8/8, 8/22, 9/10, 10/1, 10/18, 11/5
Panther Hollow Lake	40.437075, –79.948905/urban	7/18, 9/5, 11/7
Dutch Fork Lake	40.140900, –80.470901/rural	7/15, 8/13
Keystone Lake	40.373518, –79.382933/rural	8/20, 9/18
Carnegie Lake	40.480768, –79.911352/urban	8/2
Acme Lake	40.116261, –79.429153/rural	8/26
Lake Elizabeth	40.453105, –80.012007/urban	9/26

All lake water sampling procedures were performed following the Pennsylvania Department of Environmental Protection (PA DEP) protocol, with key details given here. Lake water samples were collected directly from the bloom at the water’s surface if a cyanobacterial bloom was seen or suspected. If there was no visible bloom, the lake water samples were collected in a composite manner at a spot of high interest (e.g., boat ramp or beach area). A composite water sample was collected from nine different sublocations (at the ankle, knee, and hip-depth at various zones of the water area) and then combined and mixed. Along with the 16 L water sample, an additional 250 mL water sample was collected in a similar manner and sent to PA DEP laboratories for cyanobacterial and toxin analysis.

### Lake Water Parameter Measurements

Measurements of water pH, temperature, dissolved oxygen, conductivity, total dissolved solids, and salinity were taken at the time and location of each sample collection using an Apera Instruments Premium Series Multi-Parameter Pocket Tester Kit and Milwaukee PRO Dissolved Oxygen Meter. All probes were calibrated and maintained according to the manufacturer’s specifications. Water parameter measurements for each sample can be found in the Supporting Information (SI), Table S1. Each lake sample was collected and immediately transported to Carnegie Mellon University within 1 h of collection.

Chl-a measurements were performed via UV–vis spectroscopy using a Thermo Scientific Evolution 220 UV–visible Spectrophotometer within 2 h of sample collection and are shown in Table S1.^40,41^ Chl-a concentrations were determined from 25 to 50 mL lake water samples vacuum-filtered onto 47 mm nylon filters (Whatman, 1.0 μm pore size). Filters were extracted in 25 mL 90%/10% methanol/water solvent and sonicated for 25 min. The extract was centrifuged for 30 min and measured spectrophotometrically for chl-a absorbance at 665 nm wavelength. Chl-a concentrations were determined from their absorbance using widely used relationship detailed in Lichtenthaler (1987).^42^ Chl-a concentrations correlate well with total observed cyanobacteria counts, as shown in Figure S1, with an *R* ^2^ of 0.85. As such, chl-a concentrations are assumed to represent the photosynthetic activity in the lake samples. Additionally, phycocyanin measurements were performed on seven of the lake samples via UV–vis spectroscopy. Phycocyanin concentrations correlate well with total observed cyanobacteria counts (see Figure S2) and are shown in Table S1. Phycocyanin concentration also agreed well with chl-a concentration, with an *R* ^2^ value of 0.97. Thus, this study focuses on correlating BVOC fluxes with chl-a, while phycocyanin correlation analyses are presented in the SI.

### Lake Sample Emission Measurements

After sample collection, each lake water sample was transferred to a clean 28.5 L PFA-coated stainless steel tank with an airtight flat glass top (see Figure 2). The tank was connected inline to a custom-built transverse atmospheric pressure inlet, hydronium ion high resolution CIMS.^37,43^ Before sample measurements, the tank was triple cleaned with 90% ethanol, Alconox soap detergent, and reverse osmosis (RO) water, then purged with zero air (Peak Scientific Precision Zero Air 18 L Generator) for 24 h while measuring background signals using the CIMS. Background signals did not vary significantly between samples (see Table S2) and averaged at 1 × 10^–2^ and 2.4 × 10^–1^ parts per trillion by volume (pptv) for C2-amine and pyrroline, respectively. Background concentrations of these compounds were typically 1 to 3 orders of magnitude lower than concentrations measured from water samples.

**Figure 2:**
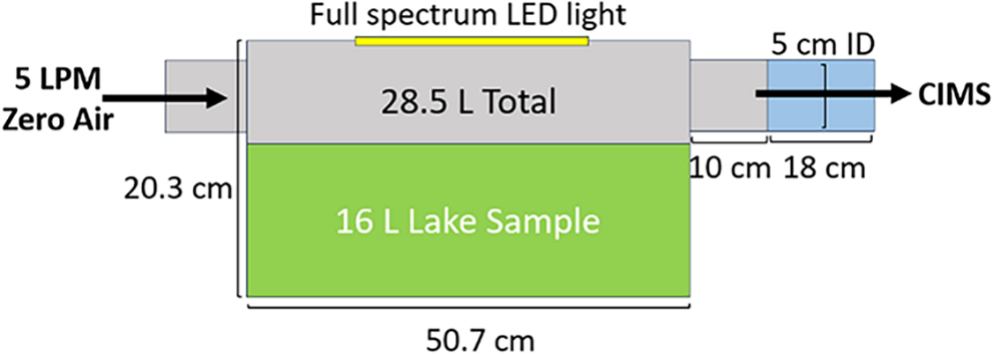
Schematic of the flux measurement tank connected inline with the CIMS inlet for evaluation of lake water sample.

Once the background signal was deemed extremely low and stable (i.e., relative standard deviation below 5%), the lake water sample was gently poured into the tank. A 14-W full-spectrum LED 12 in. light bar supplied up to 1000 μmol photons m^–2^ s^–1^ photosynthetically active radiation (PAR) above the water sample from 6:00 AM to 8:00 PM daily. Natural sunlight during peak midday may vary between 1500 to 2000 μmol photons m^–2^ s^–1^ PAR at the lake’s surface. However, sunlight exposure is highly dependent on parameters like cloud cover and shade over the water. Water samples ranged from 14 to 31 °C throughout the sampling season, with an average temperature of 21.3 °C. When sample temperatures during collection were well above room temperature (i.e., 20–22 °C), a heat mat was placed underneath the tank to maintain the water temperature at or close to the temperature of collection. As lake water temperatures can vary significantly throughout the day, replicating the diurnal variation in water temperatures was not done. Five liters per minute of zero air was passed through the tank headspace above the water sample and connected to a 18 cm long, 5 cm inner diameter glass extender coupled inline with the custom-built, transverse atmospheric pressure CIMS inlet to minimize gas losses to the walls.^43^ The average air velocity over the sample water’s surface was 2.4 × 10^–3^ m s^–1^, which is relatively low compared to typical surface velocities in the marine environment (e.g., 5 to 10 m s^–1^trade winds and open ocean). However, this value compares well with typical air velocity over lake surfaces, as wind conditions were often observed to be stagnant, though not quantified.

The CIMS operates by chemically ionizing a gaseous sample with a specific reagent ion. As many of the targeted BVOCs examined here are basic, positive polarity with reagent ions of hydronium with water ligands ((H_2_O)_0–3_·H_3_O) was used. Approximate signal intensities for H_3_O^+^, H_2_O·H_3_O^+^, (H_2_O)_2_·H_3_O^+^, and (H_2_O)_3_·H_3_O^+^ were 3e3, 5e4, 1.5e5, and 5e4 Hz, respectively. Total reagent signal was typically 2e5 Hz or higher. The measured ion signal for each ionized compound detected in the sample flow is converted to the gaseous species concentration by assuming collision rate ionization kinetics. This assumption applies well for ammonia and alkylamines,^36,44^ but likely is not the case for most BVOCs examined here. As such, concentrations and fluxes reported here represent minimum values, as a lower ionization rate coefficient would increase the converted concentration for a given observed compound signal. See SI for the ion signal to gaseous concentration conversion equations and discussion. Gaseous emissions from the water sample were continuously measured for at least 24 h after the sample was introduced to the tank. Beyond this time frame, the sample emissions are likely no longer representative of the natural gas fluxes from the lake. Since the lake sample was isolated from its natural ecosystem, it no longer interacts with other organisms and chemical components in the lake. Additionally, exposure to altered environmental conditions could influence the emission dynamics over time.

For all lake sample flux measurements, multiple ammonia (NH_3_) and water cluster peaks (i.e., NH_4_^+^, NH_3_·H_3_O^+^, NH_3_H_2_O·H_3_O^+^, NH_3_(H_2_O)_2_·H_3_O^+^) were observed in the mass spectra, thus NH_3_ concentration and flux are represented as a combination of these signals. Approximate signal intensities for 18.034 amu (NH_4_^+^), 36.044 amu (NH_3_·H_3_O^+^), 54.055 amu (NH_3_H_2_O·H_3_O^+^), and 72.065 amu (NH_3_(H_2_O)_2_·H_3_O^+^) were 5e3, 2e4, 6e3, 5e2 Hz, respectively. Methylamine (MA), C2-amine, and C3-amine signals were observed at single peaks at 32.050 amu (CH_3_NH_2_H^+^), 46.065 amu ((CH_3_)_2_NH_2_^+^), and 60.080 amu (N­(CH_3_)_3_H^+^), respectively. Masses 70.065 and 88.077 amu were determined to be 1- or 3-pyrroline (C_4_H_7_NH^+^) with a water ligand (C_4_H_7_N·H_3_O^+^), respectively. Pyrroline concentrations and fluxes are also represented as the sum of these signals. Mass 80.049 amu is suspected to be pyridine (C_5_H_5_NH^+^). Other compounds discussed (e.g., 78.055 and 124.101 amu) are represented as their measured masses only. 78.055 and 124.101 amu were included in the discussion as these peaks were in the top ten measured fluxes (see Table S3) and were consistently measured above background in all lake sample emissions. Approximately 900 unique masses were measured in all lake sample emissions where their peak signal was above background levels. These masses likely include compounds clustered with water or other compounds. However, only about 100 masses regularly maintained signals above background for the 24 h measurement period.

For comparison, duplicate baseline emission tests were conducted using 16 L samples of RO water produced by an Elga PureLab Flex 2 system, following the same procedure as the lake water emission measurements (see Figure S3). The goal was to evaluate whether the BVOCs observed in this study may also originate from clean water in the absence of biological or environmental inputs.

### Gas Concentration and Water-to-Air Flux Calculations

Lake sample emission results are reported both in gas concentration (pptv) and molar flux (mol m^–2^ day^–1^) in this work. Gas compound fluxes are calculated using the following equation:

1 $$F_{x}\;\left(moles\;m^{-2}\;day^{-1}\right)=\left(\left[X_{out}\right]-\left[X_{in}\right]\right)\times N_{A}\times \frac{Q}{A}$$

where *F*_*x*_ is the flux of the compound *X*, [*X*_out_] and [*X*_in_] are the outlet and averaged background concentrations, respectively, of the gas compound measured by the CIMS. Averaged background concentrations were taken to be the average of concentrations observed 1 h prior to a sample being introduced to the system. *N*_*A*_ is Avogadro’s number, *Q* is the volumetric flow rate through the tank headspace above the water sample, and *A* is the surface area of the water sample in the tank.

### Cyanobacteria and Toxin Screening

The additional 250 mL lake water sample at each sample collection was shipped overnight to PA DEP to perform a potentially toxigenic (PTOX) cyanobacteria screen and cyanotoxin/algal toxin analysis (see Table S1). These samples were examined using a Nikon TE200 Inverted Microscope fitted with phase contrast optics to identify specific toxin-producing cyanobacteria genera. Only samples with elevated PTOX cyanobacteria counts were further analyzed for toxins, as recommended by PA DEP. The toxins measured in the elevated PTOX cyanobacteria samples were microcystins/nodularins, cylindrospermopsin, Anatoxin-a, and saxitoxins.

Eleven out of the 18 lake samples received PTOX cyanobacteria screening, with seven analyzed for toxins. As such, chl-a concentrations were measured on all lake samples to provide a broader indicator of phytoplankton presence. Phycocyanin concentrations were measured for seven lake samples. Chl-a and phycocyanin measurements provide greater coverage of any genera not fully characterized by the PTOX screening while also providing a metric to indicate cyanobacteria presence for the samples that did not receive PTOX screening.

## Results and Discussion

### Single Experiment Concentration Time Series

Figure 3 displays the concentration time series of the Burrell Lake sample emissions on 7/24. The chl-a concentration for this sample was ∼5.4 × 10^3^ μg/L and contained a visible and concentrated cyanobacterial bloom. Stable background signals are displayed before time 0 h. Gas concentrations of all compounds shown in Figure 3 initially spike above background when the 16 L lake water sample is poured into the tank at time 0 h. Peak concentrations were typically 1 to 3 orders of magnitude higher than average background concentrations. Gas concentrations continue to either plateau or steadily decrease in signal throughout the first 24 h. All samples experienced similar time series concentration patterns for these compounds over the 24 h experiments.

**Figure 3:**
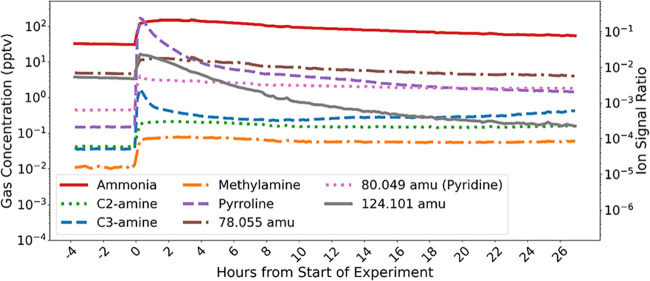
10 min averaged concentrations of various compounds observed during background and water sampling period. The water sample was collected from Burrell Lake on 7/24/24. Each color/line is a different compound. Ion signal ratio is ratio between analyte signal and total reagent signal.

C3-amine, pyrroline, and 124.101 amu exhibit a sharp initial spike in gas concentrations, reaching a maximum within 1 min of the tank being fully sealed after pouring in the sample. The concentrations then decrease by over an order of magnitude. C3-amine and pyrroline concentrations remain well above background signal, while 124.101 amu concentrations decrease below background levels over the 24 h measurement period. The rapid increase in gas concentration likely reflects the efficient transfer of these dissolved gases from the water’s surface, potentially influenced by their volatility, surface activity, or initial liquid-phase concentration in the lake water sample. C3-amine, suspected to be trimethylamine, shows higher surface activity than methylamine and C2-amine (likely dimethylamine). Surface activity, measured by a compound’s ability to reduce the surface tension of water, is greater for trimethylamine at equivalent concentrations compared to methylamine and dimethylamine.^45^ Thus, trimethylamine more readily accumulates at the water–air interface, potentially enhancing its emission into the atmosphere. Despite its high water solubility and volatility, strong surface activity implies that interfacial processes likely play a significant role in trimethylamine’s water-to-air exchange. 1- or 3-pyrroline exhibits moderate volatility, with vapor pressures at 25 °C of 0.032 and 0.072 atm, respectively. These values are substantially lower than those of the amines but are sufficient to promote rapid initial volatilization. Unlike trimethylamine, pyrroline is only moderately water-soluble, which may explain its sustained presence in the gas phase. Pyrroline’s ring structure and partial polarity may also enhance surface activity, potentially influencing water-to-air exchange rates as well. While pyrroline concentrations remain well above background, concentrations of 124.101 amu declined below background levels over the 24 h period. This trend indicates that the unknown compound may be subject to active removal from the gas phase, potentially via dissolution into the aqueous phase, with the lake water serving as a sink. However, in some lake emission measurements discussed further below, 124.101 amu concentration remained above background levels throughout the entire measurement period, indicating the emission dynamics of this compound may be dependent on more than volatility and water solubility. Determining the chemical identity of 124.101 amu will help elucidate these inconsistent emission behaviors in the future.

In contrast, ammonia, methylamine, C2-amine, 78.055 amu, and pyridine do not exhibit a large peak in concentration after the tank is sealed. Their concentration profiles quickly reach a maximum concentration within 5 min after the tank is sealed and remain relatively steady over time. This suggests that their emission dynamics are controlled by different mechanisms, potentially including continued production in the water, lower volatility, or stronger aqueous solubility.

All compounds reached relatively stable steady state concentrations within minutes of the tank being sealed with a slight decrease in concentration over time. This may be the result of a lack of biological processes, such as production or transformation by cyanobacteria, microbes, and other aquatic organisms, along with the absence of nutrient replenishment to sustain these activities. Additionally, the presence of a natural surfactant microlayer on the lake’s surface, commonly formed by organic matter and microbial exudates, may alter gas exchange rates.^20,46^ A surfactant microlayer could either suppress or selectively influence the flux of certain compounds depending on their surface activity and interactions with the microlayer, potentially contributing to the observed differences in emission patterns. However, the C3-amine concentrations approximately doubled midway through the 24 h measurement period as shown in Figure 3 (Burrell Lake 7/24), despite no changes to the experimental setup. This increase was not observed in the other lake samples. The anomalous trend for C3-amine for this single lake sample likely reflects unmeasured dynamic processes in the water, such as biological activity or variations in the surfactant layer, and highlights the complex factors influencing freshwater amine emissions. The complexities of water-to-air exchange for these compounds warrant further investigation to determine the mechanisms (e.g., emission rates, volatility, aqueous solubility, surfactant layer) governing their time-dependent emission flux patterns.

### Lake Sample Gaseous Fluxes of Ammonia and Alkylamines

Figure 4 shows the molar gas fluxes of ammonia, methylamine, C2-amine, and C3-amine from the RO water baseline and 18 lake water samples, ordered by chl-a concentrations. These flux values represent the gas emissions solely from the water samples, excluding any background contributions, as indicated in eq 1.

**Figure 4:**
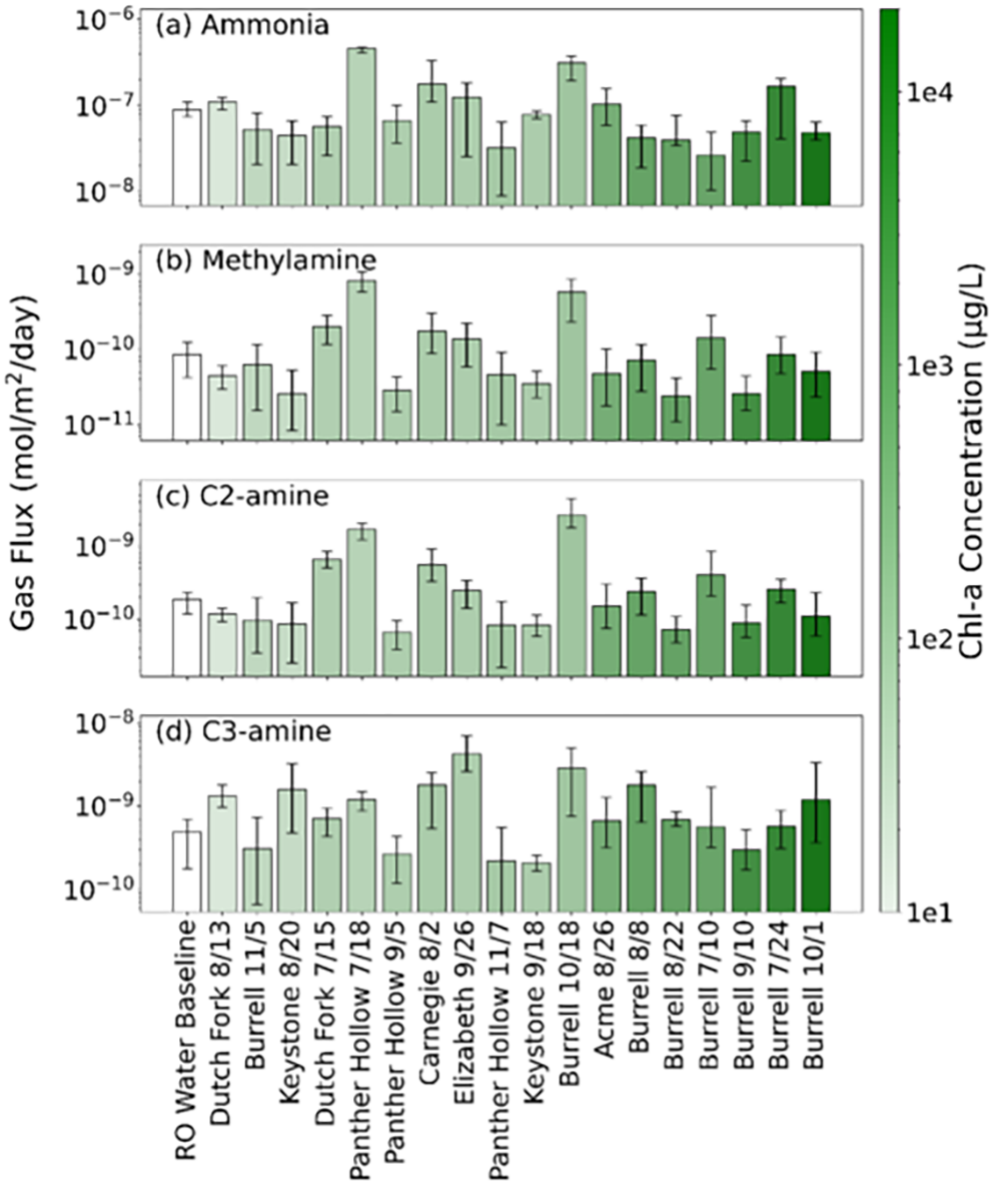
Molar gas fluxes for (a) ammonia, (b) methylamine, (c) C2-amine, and (d) C3-amine for RO water and 2024 lake samples, denoted by lake and sample collection date. The bar shows the 24 h average flux; error bars show minimum and maximum flux within the 24 h of measurements. Lake samples are ordered and colored from low to high chl-a concentration.

While chl-a concentration is commonly used to estimate total algal biomass, it does not differentiate between various algal groups, including cyanobacteria, and may not always be a reliable indicator of cyanobacteria abundance or biological activity in algal blooms.^47–49^ However, previous studies have reported positive relationships between ammonia and alkylamine emissions to algal activity in the marine environment,^19,32^ which was represented through chlorophyll pigmentation. In contrast, the results in Figure 4 indicate that there is no relationship between chl-a concentration and the observed fluxes of ammonia and alkylamines from the lake samples, as reflected by very low *R* ^2^ values (0.01 to 0.03). Additionally, the fluxes did not correlate with phycocyanin concentrations, with low *R* ^2^ values (0.01 to 0.04). Broadly, the ammonia and alkylamine fluxes do not correlate with any other measured parameter associated with each lake sample (e.g., water chemistry parameters, PTOX cyanobacteria concentration, cyanobacteria genera, lake location, or time of collection, Table S1). These observations suggest that there are likely more complex parameters that must be considered when potentially correlating ammonia and alkylamine fluxes to freshwater cyanobacterial activity.

Fluxes from the lake samples varied relative to those measured from RO water, indicating that not all observed emissions are necessarily driven by biological or environmental inputs. Approximately half of the lake samples had fluxes above the RO baseline for ammonia, methylamine, and C2-amine, while the majority of C3-amine average fluxes were higher than those from RO water. This pattern may indicate that while ammonia, methylamine, and C2-amine may have both biological and nonbiological sources, including potential background emissions from water itself, C3-amine could be more strongly influenced by microbial activity or organic matter degradation in lake systems. Previous studies have shown that trimethylamine is often the most abundant among the low molecular weight alkylamines (methylamine, dimethylamine, and trimethylamine) in both the aqueous and gas phases in aquatic environments, particularly in marine systems.^50–52^

The ammonia and alkylamine fluxes fall within 2 orders of magnitude of each other, exhibiting no clear pattern. Given the lack of trends in the fluctuations of the ammonia and alkylamine fluxes, we instead report in Table 2 a range of fluxes expected from Southwestern PA lakes during the July-November 2024 season, regardless of harmful algal bloom activity or other measured parameters.

**Table 2:** Flux Comparison between This Work and Previously Reported Ammonia and Alkylamine Fluxes^a^

compound	previously reported literature fluxes (mol m^–2^ day^–1^)^19,32^	this study (mol m^–2^ day^–1^)
ammonia	–6.5 × 10^–6^ to 2.5 × 10^–6^	8.9 × 10^–9^ to 4.8 × 10^–7^
methylamine	–1.1 × 10^–7^ to 3.5 × 10^–8^	8.4 × 10^–12^ to 1.1 × 10^–9^
C2-amine	–1.79 × 10^–7^ to 1.88 × 10^–7^	2.2 × 10^–11^ to 4.5 × 10^–9^
C3-amine	–8.1 × 10^–9^ to –5.8 × 10–9	3.7 × 10^–11^ to 7.0 × 10^–9^

a Positive flux values indicate net emission to the atmosphere, whereas negative flux values indicate uptake (deposition) from the atmosphere.

Limited studies have previously reported ammonia and alkylamine fluxes across the sea-air interface in marine environments (specifically in the tropical Atlantic Ocean and Arabian Sea).^19,32^ These fluxes are also shown in Table 2. The comparison of gaseous fluxes between previously reported values and our measurements reveals notable variations across the four compounds. Ammonia fluxes from past studies exhibit a wide range, spanning from −6.5 × 10^–6^ to 2.5 × 10^–6^ mol m^–2^ day^–1^, whereas our measured ammonia fluxes fall within a much narrower distribution. The previously reported fluxes for methylamine, C2-amine, and C3-amine span from −1.1 × 10^–7^ to 3.5 × 10^–8^, −1.79 × 10^–7^ to 1.88 × 10^–7^, and −8.1 × 10^–9^ to −5.8 × 10^–9^ mol m^–2^ day^–1^, respectively. In comparison, our observed fluxes for these compounds are significantly lower. These differences suggest that our study captures lower emission rates of ammonia and alkylamines, potentially due to variances in environmental conditions and locations, as well as measurement methods. In addition, while previous studies reported only negative fluxes of C3-amine in marine environments (i.e., deposition or uptake into the water), we observed positive fluxes of C3-amine from the freshwater lake samples. This suggests that under certain environmental conditions, such as those associated with freshwater systems influenced by biological activity, C3-amine could act as a source to the atmosphere, rather than a sink.

Water parameters (e.g., temperature, pH, and salinity) can significantly influence the volatilization of ammonia and amines. Higher temperatures generally increase volatilization rates by enhancing molecular movement. Higher pH increases the proportion of nonionized ammonia and amines, enhancing transfer to the atmosphere by shifting the equilibrium between their ionized and nonionized forms.^30,53^ An increase in salinity can also promote volatilization by reducing gas solubility, as well as altering activity coefficients and microbial nitrogen cycling.^53^ The lake water samples had pH values ranging from 7.02 to 9.05, which are generally similar to typical ocean pH levels (7.8 to 8.4). In contrast, the measured lake salinities were very low (0.12–0.33 ppt) compared to typical ocean salinities of 33–35 ppt,^54^ likely contributing to lower observed ammonia and amine emissions in these freshwater systems relative to marine studies. Even small differences in salinity can affect microbial activity and gas transfer dynamics, suggesting that freshwater and marine systems may exhibit distinct emission behaviors. Moreover, marine studies are typically conducted in the open ocean, where conditions are less affected by localized drivers such as waterfowl activity, weather-induced nutrient runoff, or seasonal biological variability that may influence freshwater systems. Additional factors, such as the composition of surfactant layers, may play a significant role in influencing ammonia and alkylamine fluxes, warranting further investigation.

Broadly, ammonia and amines are ubiquitous in the environment, originating from both anthropogenic and biogenic sources (e.g., agriculture, animal husbandry, industrial activities, microbial activity, decomposition of organic matter, etc.).^10^ In the ambient atmosphere over land, reported ammonia concentrations typically range in the parts per billion by volume (ppbv), while reported amine concentrations are generally 2 to 3 orders of magnitude lower.^10,55,56^ Thus, amine concentrations are typically between 0.01% and 1% of ammonia concentrations. Our results show that combined methylamine, C2-amine, and C3-amine concentration averaged approximately 1.5% of ammonia concentration. This ratio is higher than typically observed over land. Additionally, our results show that C3-amine concentrations averaged about 0.76% of ammonia concentrations. For comparison, trimethylamine to ammonia ratios between 0.29% and 1.02% have been reported inside animal housing facilities.^57^ This suggests that C3-amine levels in our system are comparable to those found in high-emission terrestrial environments.

One significant environmental factor that can drastically alter the water-to-air gas exchange rate is the wind speed across the water’s surface. Marine environments typically experience wind speeds ranging from 5 to 10 m s^–1^ under calm conditions, and up to 20 m s^–1^ in harsh conditions. In contrast, wind speeds over freshwater lakes are generally much lower,^58^ though they can vary significantly depending on the lake’s location, topography, and weather conditions. Higher surface velocities result in higher fluxes, which could explain the lower fluxes observed in our study. The surface velocity in the tank is 2.4 × 10^–3^ m s^–1^, which is magnitudes lower than typically observed in marine environments. Nevertheless, the observed variations between previously reported marine fluxes and our freshwater flux for ammonia and alkylamines highlight the complexity of nitrogenous gas fluxes and the need for additional studies to better understand the processes by which these gases are released into the atmosphere from water sources.

In addition, while our controlled laboratory setup allowed for careful isolation of lake-derived emissions, it does not fully replicate natural variability in environmental drivers such as diurnal temperature changes, wind speed, water column mixing, or humidity. These factors can strongly influence gas exchange dynamics through effects on water–air partitioning, microbial activity, and turbulence at the interface. As such, the flux values reported here may differ from in situ emission rates observed in the field. Moreover, as we did not directly measure particle concentrations in this study, potential consumption of ammonia and amines by new particle formation processes represents an unquantified loss pathway. This loss is not expected to be significant, as no nucleation precursor gases (e.g., sulfuric acid, methane sulfonic acid, or iodic acid) were detected with the CIMS operating with nitrate as the reagent ion in negative ion mode. Future studies should examine the potential of these emissions to form new particles from mesocosm or in situ chamber experiments. This would help constrain these uncertainties due to environmental factors, such as natural temperature and wind variability, and improve the representativeness of freshwater emission estimates.

### Lake Sample Gaseous Emissions of Heterocyclic Amines and other BVOCs

Besides ammonia and amines, the lake samples emitted many other compounds, most of which were unidentified from their measured masses. However, some higher flux compounds are shared here, and their identities are speculated. Figure 5 shows the molar gas fluxes of pyrroline (i.e., signal combination of masses 70.065 and 88.077 amu), 78.055, 80.049, and 124.101 amu from the 18 lake water samples and the RO water baseline. Note these flux values represent minimum values as the ionization rate coefficients of these compounds may be lower than the assumed ion–molecule collision limit.

**Figure 5:**
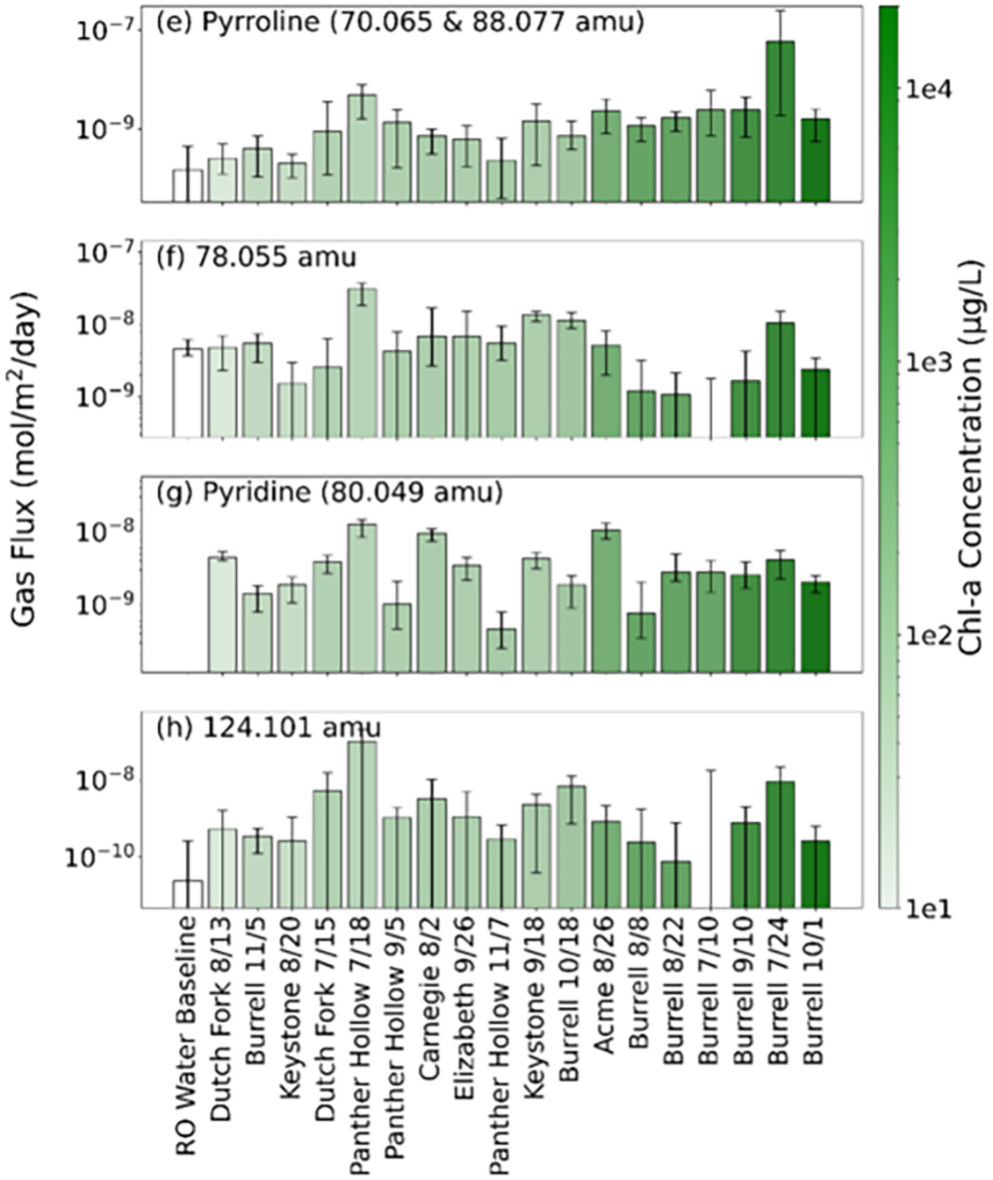
Molar gas fluxes for (a) pyrroline (70.065 and 88.077 amu), (b) 78.055 amu, (c) pyridine (80.049 amu), and (d) 124.101 amu for RO water and 2024 lake samples, denoted by lake and sample collection date. The bar shows the 24 h average flux; error bars show minimum and maximum flux within the 24 h of measurements. Lake samples are ordered from low to high chl-a concentration.

Mass 70.065 amu is proposed to be 1- or 3-pyrroline (C_4_H_7_NH^+^), and mass 88.077 amu is pyrroline with one water ligand (C_4_H_7_N·H_3_O^+^), as it perfectly correlates with 70.605 amu during the experiments. The 80.049 amu compound is suspected to be pyridine (C_5_H_5_NH^+^). Compounds 78.055 and 124.101 amu remain unidentified. The flux results presented here indicate no distinct relationship between chl-a concentration and these observed compound fluxes, with low *R* ^2^ values (0.01 to 0.09). Additionally, the fluxes did not correlate with phycocyanin concentrations, with low *R* ^2^ values (0.01 to 0.05). Like ammonia and alkylamines, the fluxes of these compounds do not correlate with any other measured parameters as well. However, the Burrell Lake sample collected on July 24 exhibited a comparatively high pyrroline flux, over a magnitude higher than the remaining samples, though the underlying reason remains unclear and is discussed further below.

Compared to the RO water average fluxes, lake water samples consistently exhibited higher pyrroline fluxes, suggesting a strong source in natural freshwater environments. Fluxes of 78.055 amu were often slightly elevated relative to RO water, indicating a possible natural source, though not significantly. In contrast, 124.101 amu showed markedly higher emissions from nearly all lake samples, averaging 1.5 × 10^–8^ mol m^–2^ day^–1^, compared to 2.5 × 10^–11^ mol m^–2^ day^–1^ in RO water, suggesting a strong, possibly biologically mediated, source. Pyridine (80.049 amu) was not detected in RO water emissions, pointing to a potentially exclusive source in lake-derived biological or chemical processes.

Nitrogen-containing heterocyclic compounds, such as pyridines, pyrrolidines, and pyrroles are well-documented in tobacco smoke and are recognized as carcinogenic.^59^ One study reported observing pyrroline in freshwater cyanobacteria emissions.^34^ Additionally, pyridine and other nitrogen-containing compounds were identified in aerosol particles sampled over the European continent and Atlantic Ocean.^60^ Despite these known sources, there have been no prior quantified reports of pyrroline or pyridine being emitted from freshwater environments. While the observed concentrations are likely too low to pose significant health risks, the unexpectedly high pyrroline fluxes from the Burrell Lake 7/24 sample underscore the complex dependencies of these emissions on water biochemical parameters. Our findings reveal that lake water acts as a previously unrecognized source of these compounds, with potential implications for atmospheric chemistry, air quality, and secondary aerosol formation.

### Untargeted Principal Component Analysis

As the above discussion focuses only on 8 targeted compounds out of 900 total compounds detected in the lake emissions, an untargeted principal component analysis (PCA) was performed on the full emission data set to determine any significant emission variations between samples. PCA is a statistical technique used to reduce the dimensionality of complex data sets while preserving key patterns and variations. It identifies the principal components (PC), linear combinations of variables that capture the most variance in the lake emission data set. Not only does the PCA determine variations across samples, but it also determines how each sample varies over time. Details on PCA methodology are described in the SI. The emission data analyzed in the PCA were converted from raw signal to background-subtracted gas concentration over the 24 h of emission measurements associated with each sample.

Figure 6a displays the first two principal components (PC1 and PC2) for all lake sample emission measurements, with each dot representing a mass spectrum at a given time during the 24 h sample experiment. Panther Hollow 7/18 and Burrell 7/24 stand out as significant outliers, while Burrell 10/18 and Dutch Fork 7/15 appear as more moderate deviations, indicating that the chemical composition of their emissions varies notably from the other samples. The remaining 14 samples form a tighter cluster, with no major differences observed, suggesting that their overall emission profiles are similar. This finding aligns with our previous observations based on ammonia, amines, and other compounds discussed earlier and reinforces the point that most sampled lake emissions exhibit minimal variation. The PCA results also show no distinct clustering of emissions of urban, suburban, or rural lake samples, indicating that emission patterns are not strongly influenced by lake location. Additional comparisons between PC1 and PC3, as well as PC2 and PC3, are shown in Figure S4, yielding results consistent with those in Figure 6.

**Figure 6:**
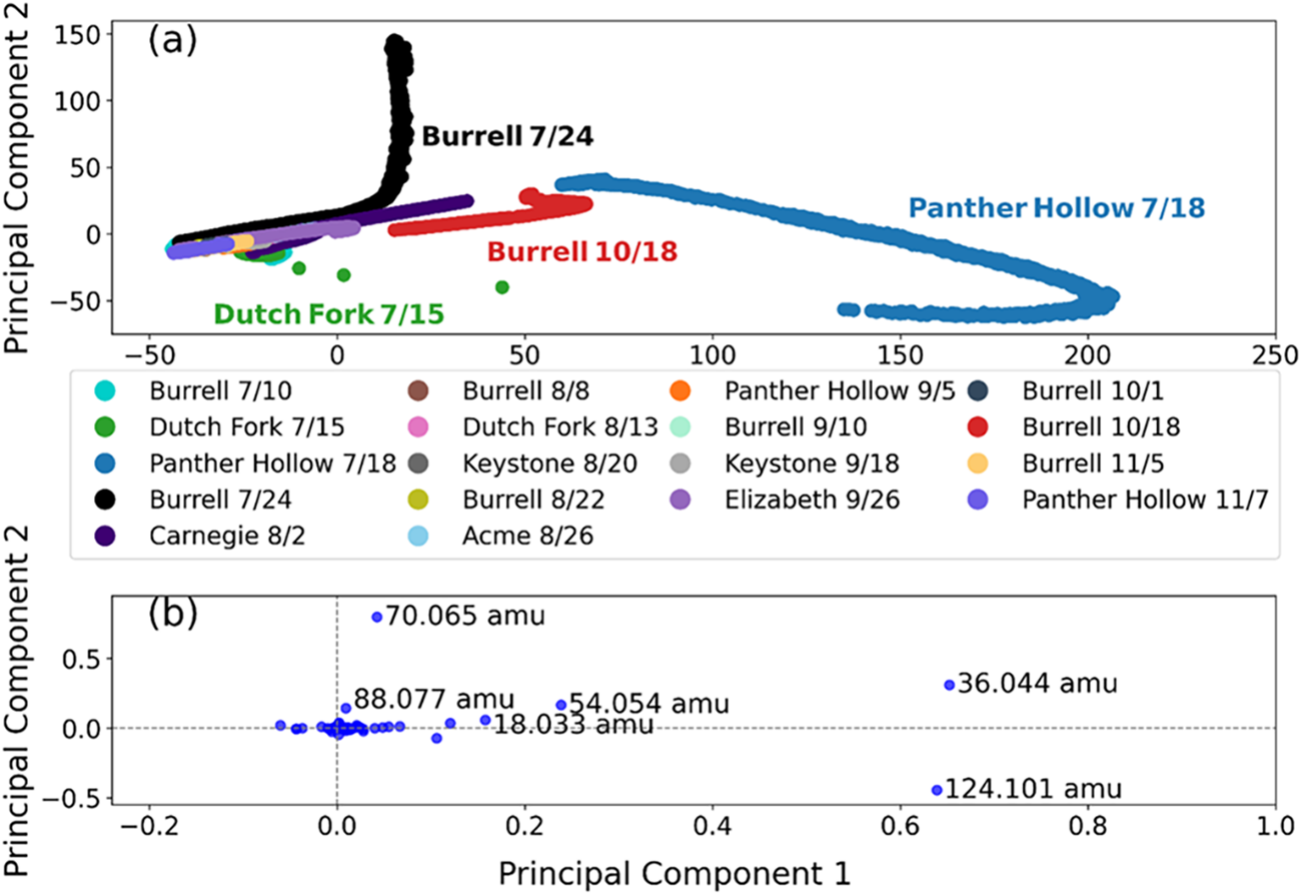
(a) Principal component 2 vs 1 for all 18 lake sample emission data, with outlier samples labeled. (b) Loading plot associated with PC2 vs PC1 with outlier masses labeled to the right of the point.

Figure 6b represents the loading plot for PC1 and PC2 comparison, which visualizes what masses are contributing to the variations of the outlier samples in the PCA. The compounds contributing to the variation for Panther Hollow 7/18 were 36.044, 54.055, and 124.101 amu. Among these, 36.044 and 54.055 amu correspond to ammonia with water ligands, which were most abundant in Panther Hollow 7/18, as shown in Figure 4. Meanwhile, 124.101 amu is an unidentified compound that is consistently detected in the lake emission mass spectra and was discussed above. Its emission patterns differ from those of ammonia and amines, as the 124.101 amu signal typically spikes sharply and then quickly returns and occasionally falls below background levels within a few hours of the sample being placed in the tank. Additionally, 124.101 amu was present in emissions from the RO water samples. However, the emission flux of 124.101 amu averaged about 3 orders of magnitude higher in lake samples compared to RO water. This indicates that lake water is a source of 124.101 amu.

Although the Panther Hollow 7/18 sample emitted higher quantities of ammonia and 124.101 amu, the reason for this increase remains unclear. The water sample parameters for Panther Hollow 7/18 were not noticeably different from those of the other samples (see Table S1). While Panther Hollow Lake resides in a valley within Pittsburgh and may experience greater or more varied runoff compared to more rural lakes, this alone does not explain the elevated emissions. Panther Hollow Lake was sampled three times in total, yet only the 7/18 sample showed an increase in these compounds. Additionally, weather conditions varied throughout the season, ranging from sunny to cloudy or foggy, with occasional light rain. However, the outlier samples, including 7/18, did not experience weather conditions that were obviously different from the other sampling days. Given these uncertainties, further investigation and additional parameter analysis are likely needed to determine the underlying factors driving the spike in ammonia and 124.101 amu in the Panther Hollow 7/18 sample.

Conversely, the variation observed in the Burrell 7/24 sample was driven by increased emissions of 70.065 and 88.077 amu, suspected to be pyrroline peaks. As with Panther Hollow 7/18, there were no significant differences in water parameters, lake location, or weather conditions that could explain the spike in these compounds. However, one study reported a sharp increase in pyrroline emission signals when a grazer was introduced to *Synechococcus elongatus*, a freshwater cyanobacterium.^34^ This suggests that the elevated pyrroline emissions in the Burrell 7/24 sample may have resulted from the presence of a grazer or another microbial dynamic interacting with the cyanobacteria. Further analysis on the microbial community is required to support this hypothesis and to better link observed emissions to the various water samples.

While no clear correlations were observed between chl-a concentrations or measured water parameters and the fluxes of the discussed BVOCs, PCA provided additional insight into both temporal and cross-lake variability. Within Burrell Lake, which was sampled eight times over the season, six emission profiles clustered closely in the PCA space, suggesting consistent chemical signatures across most sampling events. This supports the conclusion that fluxes were not strongly linked to measured water parameters even within a single lake. Temporal PCA analysis of 24 h experiments further showed that most lake samples exhibited only minor shifts in emission profiles, apart from the two outliers that were discussed. As water parameters and chl-a were measured only once per lake sample, directly linking the short-term evolution of the emission profile to dynamic changes in water chemistry is not possible. Regardless, these results underscore that standard water quality metrics such as chl-a, while widely used in atmospheric modeling, may not adequately capture the biological and chemical drivers of freshwater BVOC emissions.

## Conclusions

This study presents real-time fluxes of various BVOCs emitted from southwestern PA freshwater lake samples during peak harmful algal bloom conditions, filling a notable gap in BVOC source characterization outside terrestrial and marine environments.

Measurements of ammonia and alkylamines from the various lake samples revealed that each compound presented unique, time-dependent emission patterns rather than a uniform response throughout the sampling period. These patterns may be influenced by the compounds’ chemical properties, their interactions at the water–air interface, and other environmental or biological factors, all of which warrant further investigation. Despite using chl-a and phycocyanin concentrations, as well as PTOX cyanobacterial counts, to represent harmful algal bloom intensity, we found no clear correlations with the fluxes of ammonia, alkylamines, pyrroline, pyridine, and compounds 78.055 and 124.101 amu. This lack of correlation suggests that emission quantities and patterns are likely controlled by additional factors not measured in this work. These may include total species composition, physiological state and health of the microbial community, and nutrient levels.

These real-time observations of freshwater fluxes for ammonia, alkylamines, pyrroline, and pyridine provide valuable measurements that support more accurate atmospheric modeling. By observing the emission behavior of these nitrogenous BVOCs, our findings will improve predictions on how freshwater sources regionally influence new particle formation and secondary aerosol formation. Our results further indicate that emissions likely vary for reasons beyond the measured water parameters, highlighting the complexity of the underlying biogeochemical drivers. Further investigations incorporating species-level microbial identification and targeted metabolic assays will provide additional insight into the mechanisms driving these emissions, thereby enhancing the ability to predict the air quality and climate feedback associated with increasing HAB frequency.

## Supplementary Material



## Notes

Lake water sample parameters; correlations between chlorophyll *a*, phycocyanin, and PTOX cyanobacteria concentrations; background measurements; CIMS signal-to-concentration conversions; top fluxes from lake emissions; RO water flux measurements; and additional principal component analysis (Figures S1–S4 and Tables S1–S3) (PDF)
