# Supplementary material for: Biogenic Volatile Organic Compounds from Pennsylvanian Lakes Sampled during the 2024 Algal Bloom Season
Source: ACS EST Air. 2025 Nov 4;2(11):2603–14. doi: 10.1021/acsestair.5c00240 (PMC12624709; doi:10.1021/acsestair.5c00240)
Supplement: Supplementary file 1 [file ea5c00240_si_001.pdf]

Supplement of

Biogenic Volatile Organic Compounds from Pennsylvanian Lakes Sampled during 2024 Algal Bloom Season

Christine Troller[1,2], Dallan Schoenberger[1,2], Richard Spear[3], Jamie Detweiler[3], Jeffery Butt[4], Coty N. Jen[1,2*]

[1] Department of Chemical Engineering, Carnegie Mellon University, 5000 Forbes Ave, Pittsburgh, PA 15213, USA

[2] Center for Atmospheric Particle Studies, Carnegie Mellon University, 5000 Forbes Ave, Pittsburgh, PA 15213, USA

[3] Department of Environmental Protection, Southwest Regional Office, 400 Waterfront Drive, Pittsburgh, PA 15222, USA

[4] Department of Environmental Protection, Bureau of Clean Water, Rachel Carson State Office Building, 400 Market Street, Harrisburg, PA 17101, USA

*Email: cotyj@andrew.cmu.edu

Contents

S1. Lake Water Sample Parameters

S2. Chl-a Concentration Correlation with PTOX Cyanobacteria Concentration

S3. Phycocyanin Measurements and Correlation with PTOX Cyanobacteria Concentration

S4. Background Measurements

S5. CIMS Ion Signal to Gaseous Concentration Conversion

S6. Top Ten Fluxes Observed in Lake Sample Emissions

S7. RO Water Emission Flux Measurements

S8. Principal Component Analysis Methodology

S9. Additional Principal Component Analysis

S1. Lake Water Sample Parameters

Table S1. *Lake water sample physicochemical conditions, chl-a, phycocyanin, PTOX cyanobacteria screen, and toxin measurements.*

Collection Date & Time 24H	Lake	Water temp (℃)	pH	DO[1] (mg/L)	Cond[2] (µS/cm)	TDS[3] (ppm)	Sal[4] (ppt)	chl-a (µg/L)	PC[5] (µg/L)	PTOX Cyanobacteria Screen (counts/mL)	Toxin Analysis[6]
7/10/24 12:00	Burrell Lake	31.0	9.03	12.0	311	221	0.16	926	NA	Microcystis 1000	M BDL
7/15/24 10:30	Dutch Fork Lake	25.3	8.05	6.4	NA	NA	NA	75.4	NA	Aphanizomenon 14<br>Aphanocapsa 29<br>Dolichospermum 14<br>Pseudanabaena 560	M BDL
7/18/24 10:00	Panther Hollow Lake	24.4	7.23	0.9	565	403	0.28	62.9	520	Aphanocapsa 29<br>Pseudanabaena 110<br>Dolichospermum 14	NA
7/24/24 11:00	Burrell Lake	24.2	8.65	7.2	279	271	0.18	5402	11600	Microcystis 43000<br>Planktolyngbya 10	M BDL
8/2/24 10:00	Lake Carnegie	26.6	7.20	3.3	396	314	0.20	94.5	1190	NA	NA
8/8/24 9:45	Burrell Lake	24.4	8.65	7.3	332	233	0.12	1013	6630	Microcystis 6900<br>Aphanizomenon 1000	M, A, S, C<br>BDL

8/13/24	Dutch Fork Lake	23.2	7.69	5.42	NA	NA	NA	18.3	1520	Planktolyngbya 340<br>Aphanocapsa 130<br>Pseudanabaena 57<br>Raphidiopsis 95<br>Chroocococcus 76	NA
8/20/24	Keystone Lake	NA	NA	NA	NA	NA	NA	37.7	NA	NA	NA
8/22/24 9:45	Burrell Lake	20.7	8.61	9.0	251	259	0.18	1038	NA	Aphanizomenon 2100<br>Microcystis 150	NA
8/26/24 10:45	Acme Lake	22.5	7.41	4.1	295	211	0.15	572	NA	Aphanizomenon 10000<br>Microcystis 40	M BDL
9/5/24 9:45	Panther Hollow Lake	17.8	7.60	3.5	588	441	0.31	83.6	NA	NA	NA
9/10/24 9:45	Burrell Lake	19.0	7.96	7.5	259	258	0.18	3380	13140	Microcystis 18000<br>Aphanizomenon 2900	A 0.98 ug/L,<br>M S, C BDL
9/18/24 8:30	Keystone Lake	22.0	7.33	7.8	NA	NA	NA	90.7	NA	NA	NA
9/26/24 9:15	Lake Elizabeth	22.2	9.05	7.7	445	303	0.22	88.7	NA	NA	NA
10/1/24 10:30	Burrell Lake	19.4	7.80	3.7	373	264	0.18	14790	50500	Microcystis 160000<br>Aphanizomenon 870	A 0.91 ug/L,<br>M 3.74 ug/L
10/18/24 10:15	Burrell Lake	12.8	8.47	9.8	403	287	0.20	139.1	NA	NA	NA
11/5/24 9:15	Burrell Lake	14.4	8.38	6.7	412	294	0.21	46.8	NA	Microcystis 110	NA
11/7/24 9:00	Panther Hollow Lake	14.9	8.06	7.6	661	468	0.33	111	NA	NA	NA

[1] Dissolved Oxygen, [2] Conductivity, [3] Total Dissolved Solids, [4] Salinity, [5] Phycocyanin

[6] M: Microcystins, A: Anatoxin-a, S: Saxitoxin, C: Cylindrospermopsin, BDL: Below detection limit

Table S1 presents the date and time of collection and physicochemical parameters of lake water samples measured during this study. These values provide context for interpreting biogeochemical and microbial processes across lake samples. Chl-a and phycocyanin concentrations serve as cyanobacteria biomass indicators, while other characteristics (e.g., temperature, pH, DO) reflect conditions that influence biological activity and gas solubility. While these measurements captured environmental variation among lake samples, no consistent or significant correlations were observed between gas emissions and any individual parameter.

S2. Chl-a Concentration Correlation with PTOX Cyanobacteria Concentration

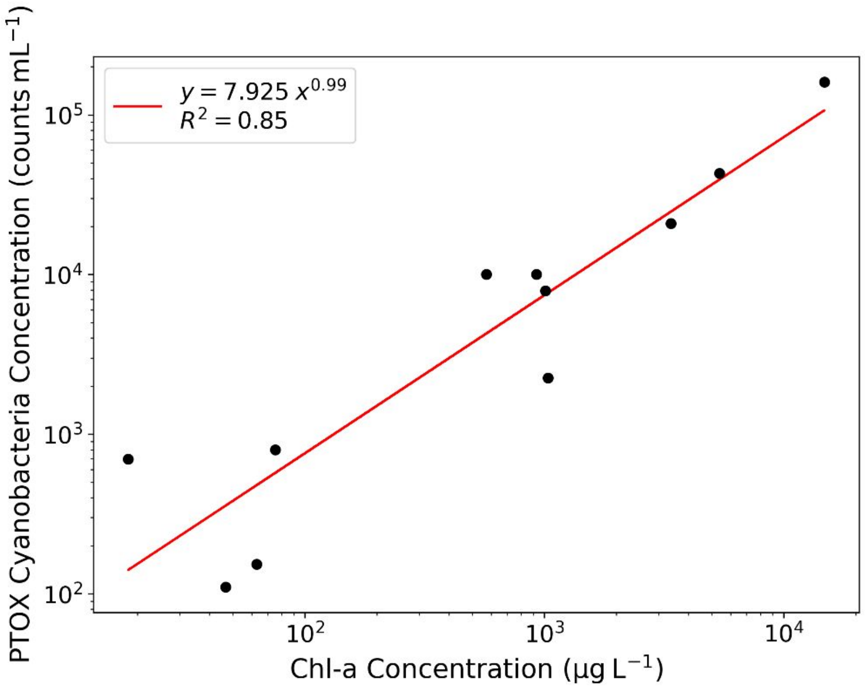

Figure S1. *Chl-a concentration and total PTOX cyanobacteria concentration correlation.*

Figure S1 shows the relationship between chl-a concentration, calculated using chl-a conversion equations from Lichtenthaler (1987),[1] and total cyanobacterial concentration, determined by PTOX screening for 11 lake water samples. Both variables are plotted on a logarithmic scale, revealing a strong positive corrleation with an R^2 of 0.85. These results support the use of chl-a as an indicator for PTOX cyanobacterial presence in the lake water samples.

S3. Phycocyanin Concentration Measurements and Correlation with PTOX Cyanobacteria Concentration

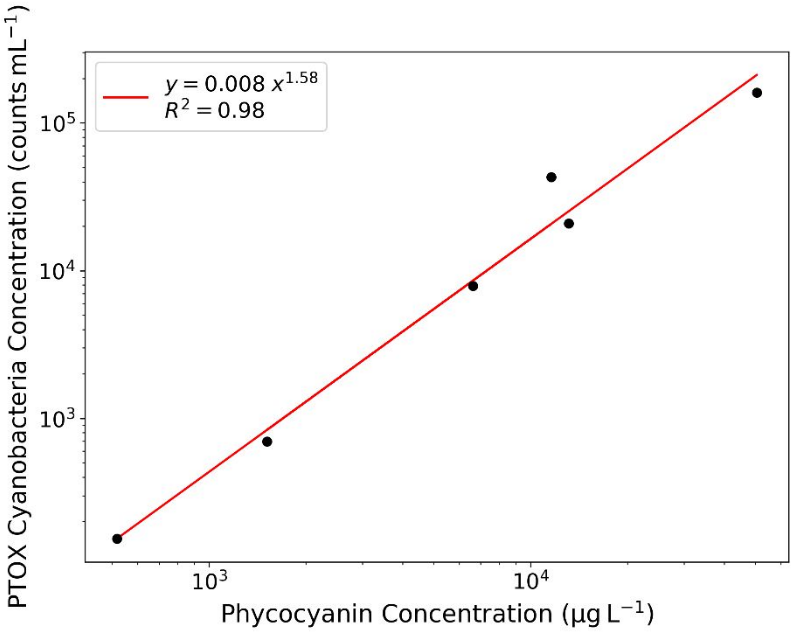

Figure S2. *Phycocyanin concentration and total PTOX cyanobacteria concentration correlation.*

Phycocyanin concentrations were shown to correlate well with the PTOX screening cyanobacteria counts for six lake samples that received both phycocyanin measurements and PTOX cyanobacteria screening, as shown in **Figure S2**. Phycocyanin measurements were performed using a Thermo Scientific Evolution 220 UV-Visible Spectrophotometer (**Table S1**). Phycocyanin concentrations were determined from 10 to 50 mL lake water samples vacuum-filtered onto 47 mm nylon filters (Whatman, 1.0 μm pore size). These filters were stored at -4℃ for at least 3 months, then thawed for 24 hours. One freeze-thaw cycle was deemed to be sufficient, as additional cycles did not exhibit greater absorbance. Each filter was submerged in 25 mL of RO water and sonicated for 20 minutes. 15 mL of the resuspended solution was then centrifuged for 25 minutes. Absorbance measurements were taken at 620 nm with a path length of 1 cm.[2] Conversion equations from Bennett and Bogorad (1973) were used to convert absorbances to concentration.[3]

S4. Background Measurements

Table S2. *Background flux measurements. Flux values are in mol m^{-2} day^{-1}.*

Compound	Overall Background Flux Mean	Overall Background Flux Std Dev
Ammonia	2.4×10^{-08}	1.5×10^{-08}
32.050 amu (MA)	2.4×10^{-11}	2.1×10^{-11}
46.065 amu (C2-Amine)	1×10^{-10}	1.5×10^{-10}
60.080 amu (C3-Amine)	1.1×10^{-10}	8.3×10^{-11}
70.065 amu (Pyrroline)	3.1×10^{-10}	1.6×10^{-10}
78.055 amu	4.4×10^{-09}	2.5×10^{-09}
80.049 amu (Pyridine)	7.5×10^{-10}	1.4×10^{-10}
124.101 amu	1.6×10^{-09}	1.8×10^{-09}

Table S2 shows the overall average background fluxes and associated standard deviations of targeted compounds discussed in this work. Background signals were measured for 24 hours from the clean, empty tank prior to all lake sample emission measurements. While background levels varied moderately across experiments, they remained consistently lower than the corresponding sample measurements. This provides confidence that observed emission patterns from the lake water samples reflect biological or chemical sources rather than fluctuations in background conditions.

S5. CIMS Ion Signal to Gaseous Concentration Conversion

The measured ion signal (Hz) for each mass detected in the sample flow is converted to the gaseous species concentration by assuming the following elementary chemical ionization reaction: $X + (H_2O)_n \bullet H_3O^+ \rightarrow HX^+ + (H_2O)_n$. This reaction occurs at the ion-molecule collision limit, k, which is taken to be 2 x 10^{-9} cm^3 s^{-1}.[4–6] The reagent ion concentration is assumed to be high and thus does not change with time. Previous studies have shown that the concentration of analyte ion ([X]) does not change by more than 5% during the short chemical ionization process.[7] The CIMS signal is then directly proportional to gas concentration and the chemical ionization reaction time. The chemical ionization time depends on the plate distance, which refers to the distance between the front and back of the inlet where decreasing voltages are being applied, the ion mobility, and the electric field. The following equations are used to convert the analyte ion signal, S, to analyte concentration [X]:

$$t_{CI}\left(chemical\ ionization\ reaction\ time\ [s]\right) = \frac{plate\ distance\ (cm)}{ion\ mobility\ \left(\frac{cm^2}{V*s}\right) x\ E\ \left(\frac{V}{cm}\right)} \quad \textbf{Equation (S1)}$$

$$[\boldsymbol{X}]\ molcules/cm^3 = \frac{\boldsymbol{S_X}}{\boldsymbol{S_{19}+S_{37}+S_{55}+S_{73}}}\boldsymbol{MDTE}\left(\frac{\boldsymbol{1}}{\boldsymbol{kt_{CI}}}\right) \quad \textbf{Equation (S2)}$$

Equation (S2) contains a ratio of different signals, where S_x is the signal for the analyte, S_{19} is the signal for H_3O^+, S_{37} is the signal for $H_2O{\bullet}H_3O^+$, S_{55} is the signal for $(H_2O)_2{\bullet}H_3O^+$, S_{73} is the signal for $(H_2O)_3{\bullet}H_3O^+$.

Mass-dependent transmission efficiency (MDTE) refers to the variation in how effectively a mass spectrometer transmits ions of different mass-to-charge ratios to the detector, which introduces bias into concentration calculations if ions of differing masses are not transmitted equally.[8] Accounting for MDTE is important when comparing signals across a wide mass range. Thus, signals for all masses observed in this study are corrected from their respective MDTE, with values extrapolated from Heinritzi et al. (2016).[8]

Additionally, not all compounds are ionized at the ion-molecule collision limit in CIMS, particularly when using hydronium ($(H_2O)_n{\bullet}H_3O^+$) reagent ions at atmospheric pressure. Ionization efficiency depends on molecular properties such as proton affinity, polarity, and structure.[9] However, for ammonia and amines, prior studies and our calibration following Fomete et al. and Freshour et al. have shown that ionization does indeed occur at the collision limit.[6,10,11] Therefore, the reported concentrations and fluxes for these species are considered accurate within experimental uncertainty. The fluxes and concentrations of the other compounds reported represent the lower limit estimate, as the ionization rate coefficients cannot exceed the collision limit. Future work should identify and calibrate these compounds to determine their overall ionization rate constant with ($(H_2O)_n{\bullet}H_3O^+$) reagent ions.

S6. Top Ten Fluxes Observed in Lake Sample Emissions

Table S3. *Top ten peaks with highest lake emission fluxes measured from lake samples, in order from highest to lowest average flux. Flux values are in mol m^{-2} day^{-1}.*

Compound	Potential Chemical Formula	Average Flux	Minimum Flux	Maximum Flux
36.044 amu	NH3H3O⁺	8.1×10^{-08}	5.4×10^{-09}	2.9×10^{-07}
54.055 amu	NH3H2OH3O⁺	3.0×10^{-08}	6.0×10^{-10}	9.7×10^{-08}
18.034 amu	NH4⁺	2.2×10^{-08}	0	8.9×10^{-08}
124.101 amu	NA	1.5×10^{-08}	0	2.0×10^{-07}
70.065 amu	C4H7NH⁺	1.0×10^{-08}	3.8×10^{-11}	2.5×10^{-07}
78.055 amu	Unknown	8.1×10^{-09}	0	3.7×10^{-08}
80.049 amu	C5H5NH⁺	4.8×10^{-09}	2.5×10^{-10}	1.5×10^{-08}
72.065 amu	NH3(H2O)2H3O⁺	2.7×10^{-09}	4.3×10^{-13}	1.0×10^{-08}
29.998 amu	Unknown	2.3×10^{-09}	0	4.4×10^{-08}
88.077 amu	C4H7NH3O⁺	1.9×10^{-09}	0	3.9×10^{-08}

Table S3 presents the ten peaks with the highest fluxes observed across all 18 lake samples. Reagent peaks, as well as peaks exhibiting poor peak fits, were excluded from this selection. Fluxes of ammonia and alkylamines represent true values, while the fluxes of other compounds represent the minimum flux value due to the assumed ion-molecule collision limit. Ammonia with water ligand clusters, at 36.044 amu, 54.055 amu, and 18.034 amu, were overall the top three highest fluxes measured in all lake sample emissions, with average fluxes being 8.1 x 10^{-8}, 3.0 x 10^{-8} mol m^{-2} day^{-}1, and 2.2 x 10^{-8} mol m^{-2} day^{-1}, respectively. The 72.065 amu

ammonia peak was the eighth highest flux at 2.7 x 10^{-9} mol m^{-2} day^{-1}. Additional top ten flux compounds are discussed in the main paper, apart from compound 29.998 amu. This compound appeared in most lake sample emissions, but did not appear in emissions from RO water samples (see **Figure S3**). Further analysis using other measurement techniques, such as two-dimensional gas chromatography coupled to a mass spectrometer, is required to better elucidate the chemical identities of the unidentified compounds observed in these emissions.

S7. RO Water Emission Flux Measurements

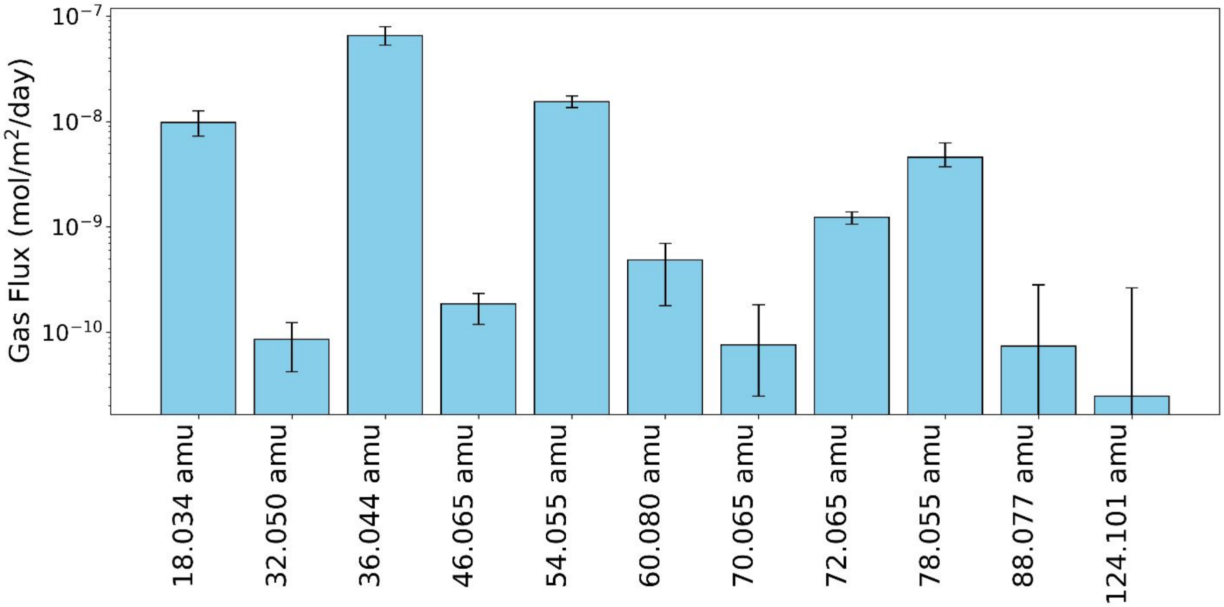

Figure S3. *Average fluxes for the alkylamines and compounds listed in Table S3, with minimum and maximum estimated fluxes shown in the bar lines (minimum is 0 if bar line hits the x-axis) of measured from RO water.*

Figure S3 shows the measured emission fluxes of the discussed compounds and compounds listed in **Table S3** from RO water using the same sampling procedures as the lake water samples. Performing this control measurement helps to identify potential emission sources that may not originate from biological presence or lake water. Compounds detected in RO water were also among the top fluxes reported in lake samples (as shown in **Table S3**), indicating that a subset of these emissions may originate from water. Note that compounds 80.049 amu (pyridine) and 29.998 amu were not detected in the mass spectra for RO water emissions. While the overall fluxes from RO water were lower than those from lake samples, some overlap of fluxes highlights the importance of accounting for this RO water test when interpreting lake emission data. These RO emissions are included in **Figures 4** and **5** to provide context and confirm that reported lake water fluxes exceed RO water background levels, indicating that lake water is an elevated source of these compounds.

S8. Principal Component Analysis Methodology

PCA was performed on the untargeted mass spectral concentration time series from each lake sample emission experiment. For each sample, the background-subtracted, 24-hour gas concentration observations across the measured *m/z* values were used as input. Data matrices from all lake samples were concatenated and subjected to dimensionality reduction using the scikit-learn Python package (*sklearn.decomposition.PCA*). All data were mean-centered prior to decomposition. PCA was performed with three components. The PC1 vs. PC2 scores plot and corresponding loading plot are presented in **Figure 6**, while PC1 vs. PC3 and PC2 vs. PC3 plots are given below in **Figure S4** to confirm the identification of two significant outlier samples.

S9. Additional Principal Component Analysis

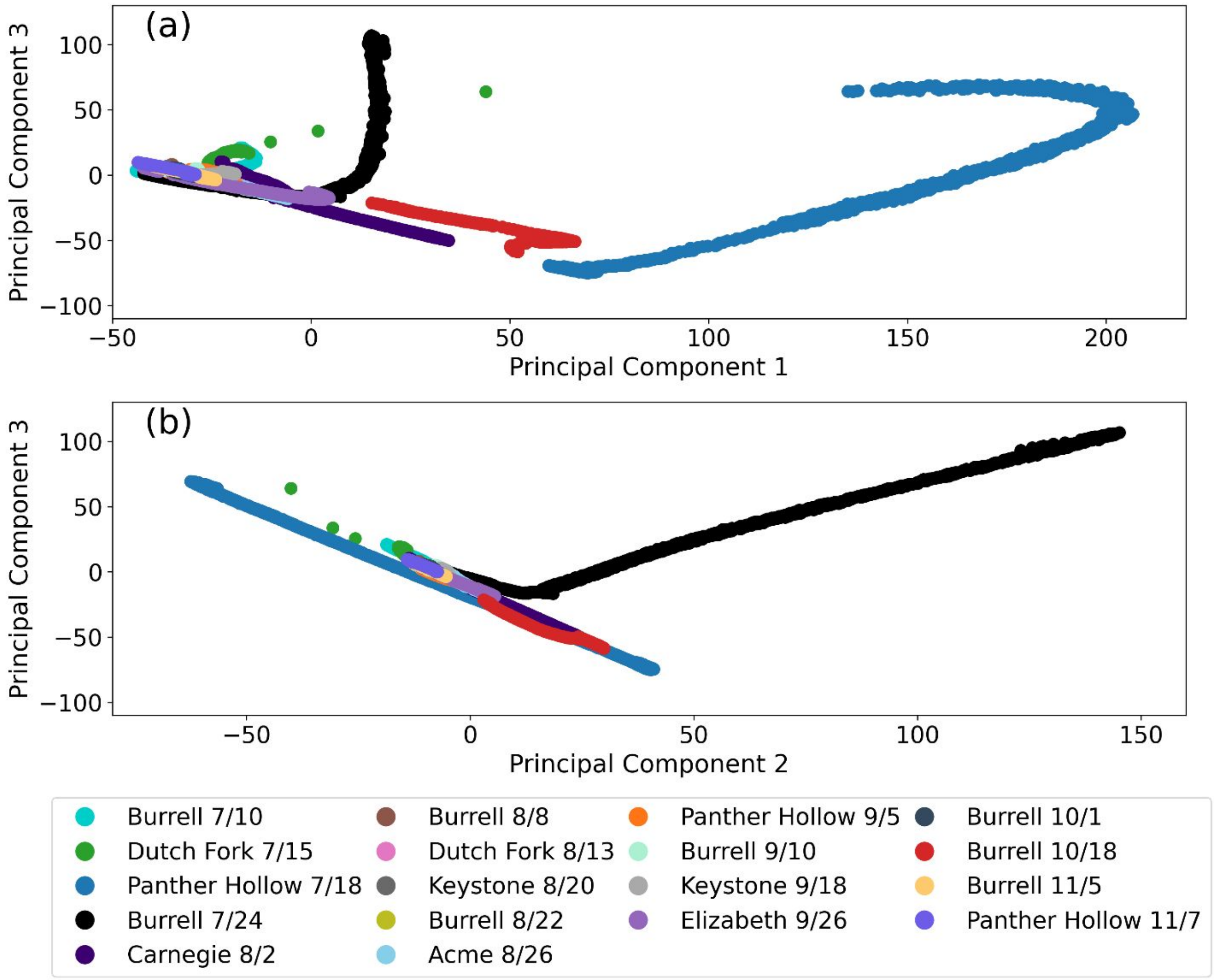

Figure S4. *Principal component analysis comparisons of additional components. (a) PC1 vs. PC3. (b) PC2 vs PC3*

Figure S4, a comparison between PC1 and PC3, as well as PC2 and PC3, results in the same conclusion represented by the comparison between PC1 and PC2. Panther Hollow 7/18 (blue dots) and Burrell 7/24 (black dots) are the two signficant outliers, with the same compounds contributing to these variations. The additional component combinations help validate the patterns observed in the primary PCA, particularly confirming the identity and separation of outlier samples. No substantial new clustering or trends were revealed along PC3, indicating that most of the meaningful variation across samples is captured within the first two principal components. This supports the interpretation that PC1 and PC2 effectively summarize the dominant sources of variability in the emission dataset.

References

(1) Lichtenthaler, H. K. [34] Chlorophylls and Carotenoids: Pigments of Photosynthetic Biomembranes. In *Plant Cell Membranes*; Methods in Enzymology; Academic Press, **1987**; Vol. 148, pp 350–382. https://doi.org/10.1016/0076-6879(87)48036-1.

(2) Khandual, S.; Sanchez, E. O. L.; Andrews, H. E.; de la Rosa, J. D. P. Phycocyanin Content and Nutritional Profile of Arthrospira Platensis from Mexico: Efficient Extraction Process and Stability Evaluation of Phycocyanin. *BMC Chem.* **2021**, *15* (1), 24. https://doi.org/10.1186/s13065-021-00746-1.

(3) Bennett, A.; Bogorad, L. COMPLEMENTARY CHROMATIC ADAPTATION IN A FILAMENTOUS BLUE-GREEN ALGA. *J. Cell Biol.* **1973**, *58* (2), 419–435. https://doi.org/10.1083/jcb.58.2.419.

(4) Španěl, P.; Smith, D. Selected Ion Flow Tube Studies of the Reactions of H3O+, NO+, and O2+ with Several Amines and Some Other Nitrogen-Containing Molecules. *Int. J. Mass Spectrom.* **1998**, *176* (3), 203–211. https://doi.org/10.1016/S1387-3806(98)14031-9.

(5) Su, Timothy.; Bowers, M. T. Ion-Polar Molecule Collisions. Effect of Molecular Size on Ion-Polar Molecule Rate Constants. *J. Am. Chem. Soc.* **1973**, *95* (23), 7609–7610. https://doi.org/10.1021/ja00804a011.

(6) Hanson, D. R.; Mcmurry, P. H.; Jiang, J.; Jiang, J.; Tanner, D. J.; Huey, L. G. Ambient Pressure Proton Transfer Mass Spectrometry: Detection of Amines and Ammonia. *Environ. Sci. Technol.* **2011**, *45 20*, 8881–8888.

(7) Berresheim, H.; Elste, T.; Plass-Dülmer, C.; Eiseleb, F. L.; Tannerb, D. J. Chemical Ionization Mass Spectrometer for Long-Term Measurements of Atmospheric OH and H2SO4. *Int. J. Mass Spectrom.* **2000**, *202* (1), 91–109. https://doi.org/10.1016/S1387-3806(00)00233-5.

(8) Heinritzi, M.; Simon, M.; Steiner, G.; Wagner, A. C.; Kürten, A.; Hansel, A.; Curtius, J. Characterization of the Mass-Dependent Transmission \hack\newline Efficiency of a CIMS. *Atmospheric Meas. Tech.* **2016**, *9* (4), 1449–1460. https://doi.org/10.5194/amt-9-1449-2016.

(9) Yuan, B.; Koss, A.; Warneke, C.; Gilman, J. B.; Lerner, B. M.; Stark, H.; de Gouw, J. A. A High-Resolution Time-of-Flight Chemical Ionization Mass Spectrometer Utilizing Hydronium Ions (H_3O^+ ToF-CIMS) for Measurements of Volatile Organic Compounds in the Atmosphere. *Atmospheric Meas. Tech.* **2016**, *9* (6), 2735–2752. https://doi.org/10.5194/amt-9-2735-2016.

(10) Freshour, N. A.; Carlson, K. K.; Melka, Y. A.; Hinz, S.; Panta, B.; Hanson, D. R. Amine Permeation Sources Characterized with Acid Neutralization and Sensitivities of an Amine Mass Spectrometer. *Atmospheric Meas. Tech.* **2014**, *7* (10), 3611–3621. https://doi.org/10.5194/amt-7-3611-2014.

(11) Fomete, S. K. W.; Johnson, J. S.; Casalnuovo, D.; Jen, C. N. A Tutorial Guide on New Particle Formation Experiments Using a Laminar Flow Reactor. *J. Aerosol Sci.* **2021**, *157*, 105808. https://doi.org/10.1016/j.jaerosci.2021.105808.
